# Evidence of this speculation is needed before the possible beneficial effect of the ketogenic diet can be attributed to the increase in wild‐type mtDNA

**DOI:** 10.1111/cns.14417

**Published:** 2023-08-21

**Authors:** Josef Finsterer

**Affiliations:** ^1^ Neurology and Neurophysiology Center Vienna Austria

We read with interest the article by He et al. on a 20‐year‐old woman with mitochondrial encephalopathy, lactic acidosis, and stroke‐like episodes (MELAS) syndrome due to the variant m.3243A>G (heteroplasmy rate 55%) who benefited from the ketogenic diet (KD) over a period of 3 years with a break after 2.7 years.^1^ Features that improved over this period included severity and frequency of seizures, frequency of stroke‐like episodes (SLEs), headache, vision, hearing, gastrointestinal symptoms, and exercise intolerance.^1^ It was concluded that KD should not be interrupted but continued for life.^1^ The study is compelling but has limitations that should be discussed.

A limitation of the study is the design. Since the validity of case reports is limited, the results cannot be easily generalized and must be confirmed by randomized and controlled studies. Although other case studies have also shown a positive effect of KD in MELAS,^2^ it still remains uncertain whether the positive effect is random or generalizable. Therefore, it is desirable that these preliminary results be confirmed by more substantiated studies on large, homogenous cohorts.

The authors speculate that improvement in the index patient was due to an increase in the amount of wild‐type mtDNA but do not provide any evidence that the amount of wild‐type mtDNA really increased in the index patient during the KD.

Compliance with KD was monitored by measuring serum levels of ketone bodies. Figure 2 shows 43 measurements, i.e. about one per month.^1^ Did the clinical monitoring also ensure that the patient was complying with the KD in the 4 weeks prior to blood collection?

Because the reference limits for serum ketone bodies are missing, it cannot be assessed whether the reported values were within or outside the normal range throughout the KD.

The patient experienced a fifth SLE coinciding with discontinuation (non‐compliance) of KD at 2.7 years after initiation.^1^ This suggests that stopping KD may trigger the development of SLE. We should know whether KD cessation was indeed temporarily linked to the fifth SLE and what alternative triggers of the fifth SLE, such as seizures, infection, psychological or physical stress, or new medications, have been ruled out.

For seizures, the patient had been on oxcarbazepine since she was 13 and multiple anti‐seizure drugs (ASDs) since she was 16.^1^ Because some of the ASDs can be mitochondrion‐toxic, particularly barbiturates, phenytoin, valproate, carbamazepine, and zonisamide,^3^ it is important to know which ASDs were actually administered and what dosage. It should also be discussed whether reducing ASDs after initiation of KD contributed to the positive effect of KD. It should also be mentioned whether low or high‐calorie KD was administered.

The patient took coenzyme‐Q and L‐carnitine during the time on the KD.^1^ How could it be ruled out that these drugs were effective and not the KD?

An improvement in non‐epileptic symptoms due to KD has been previously reported, even in animal models,^4^ and is therefore not a new observation as claimed by the authors.

Overall, the interesting study has limitations that put the results and their interpretation into perspective. Addressing these issues would strengthen the conclusions and could improve the status of the study. Although there is evidence from case reports that KD may have an anti‐seizure effect and may be beneficial in other manifestations of mitochondrial disorders (MIDs), randomized, controlled trials are desirable to confirm or rule out a long‐term beneficial effect of KD on all phenotypic features of MIDs,

## FUNDING INFORMATION

Financial disclosures for the previous 12 months: the authors declare that the research was conducted in the absence of any commercial or financial relationships that could be construed as a potential conflict of interest.

## AUTHOR CONTRIBUTIONS

Author Josef Finsterer: Research project: A. Conception, B. Organization, C. Execution; Manuscript: A. Writing of the first draft, B. Review and Critique.

## CONFLICT OF INTEREST STATEMENT

None.

## Data Availability

The data that support the findings of this study are available from the corresponding author upon reasonable request.
